# A comprehensive transcriptomic analysis of alternate interferon signaling pathways in peripheral blood mononuclear cells in rheumatoid arthritis

**DOI:** 10.18632/aging.203432

**Published:** 2021-08-25

**Authors:** Liang Han, Shenghao Tu, Pan Shen, Jiahui Yan, Yao Huang, Xin Ba, Tingting Li, Weiji Lin, Huihui Li, Kun Yu, Jing Guo, Ying Huang, Kai Qin, Yu Wang, Zhe Chen

**Affiliations:** 1 Department of Integrated Traditional Chinese and Western Medicine, Tongji Hospital of Tongji Medical College of Huazhong University of Science and Technology, Wuhan 430030, China; 2 Department of Cardiology, Tongji Hospital of Tongji Medical College of Huazhong University of Science and Technology, Wuhan 430030, China; 3 Wuhan Institute of Biotechnology, Wuhan Biobank, Wuhan 430000, China

**Keywords:** rheumatoid arthritis (RA), type I interferon, interferon-γ (IFN-γ), single-cell sequencing (SCS), peripheral blood mononuclear cells (PBMCs)

## Abstract

Interferon (IFN) signaling pathways play crucial roles in the pathogenesis of rheumatoid arthritis (RA). Prior studies have mainly studied mixed alterations in the IFN signaling pathway in RA, but these studies have not been sufficient to elucidate how imbalanced IFN signaling subtly influences immune cells. Single-cell RNA (scRNA) sequencing makes it possible to better understand the alternations in the interferon signaling pathways in RA. In the present study, we found that IFN signaling pathways were activated in natural killer (NK) cells, monocytes, T cells, B cells, and most immune cell subclasses in RA. We then explored and analyzed the connections between abnormal IFN signaling pathways and cellular functional changes in RA. Single-Cell rEgulatory Network Inference and Clustering (SCENIC) analysis and gene regulatory network (GRN) construction were also performed to identify key transcription factors in RA. Finally, we also investigated altered IFN signaling pathways in multiple RA peripheral blood samples, which indicated that abnormal IFN signaling pathways were universally observed in RA. Our study contributes to a better understanding of the delicate and precise regulation of IFN signaling in the immune system in RA. Furthermore, common alternations in IFN signaling pathway-related transcription factors could help to identify novel therapeutic targets for RA treatment.

## INTRODUCTION

Rheumatoid arthritis (RA) is a systemic, chronic, incurable autoimmune inflammatory disease affecting approximately 0.5%-1% of the world population [1]. Imbalanced immune responses both in circulating peripheral blood and in diseased joint cavities are closely related to the occurrence and development of RA [2]. It has been repeatedly illustrated that aberrant CD4^+^ helper T (Th) cells produce proinflammatory cytokines, and abnormally activated B cells can differentiate into plasma cells that ultimately produce a large panel of autoantibodies [3, 4]. In addition, aberrant alterations of innate immune cells such as monocytes, macrophages, and natural killer (NK) cells, as well as dendritic cells (DCs), are also related to RA [5–7]. Interferons (IFNs) are a family of cytokines produced by various cells that play pivotal roles in early defense against viral infection in mammals [8, 9]. Moreover, as important immunomodulators, IFNs also impressively affect several immunity responses [10]. Both abnormal levels of IFNs and alterations in IFN signaling pathways have been observed in RA, systemic lupus erythematosus (SLE), primary Sjögren syndrome (pSS), and other autoimmune diseases [11–13]. However, the specific mechanisms of IFN signaling pathways in RA are poorly understood.

Although the etiology and pathogenesis of RA are still not fully understood, it has been demonstrated that IFN- and IFN-related signaling pathways partly promote the inflammatory response in RA patients [13]. Human IFN can be broadly grouped into three main classes: type I IFN, type II IFN, and type III IFN [14]. type I IFNs consists of IFN-α, IFN-β, IFN-ε, IFN-κ, and IFN-ω, and type II IFNs consists of only IFN-γ [15]. Type III IFN was discovered approximately two decades ago and includes IFN-λ1, IFN-λ2, IFN-λ3 as well as IFN-λ4 [16]. In our study, we primarily discuss type I IFN (IFN-α and IFN-β) and type II IFN due to their important roles in RA.

Individual case reports showed that patients were diagnosed with RA after using IFN-α to treat other diseases [17, 18]. Previous studies have also reported that type I IFN response genes are potential biomarkers for predicting the development of RA [11, 19]. The relationship between IFNs and RA is not only restricted to high clinical relevance but is also reflected in many molecular biology studies. IFN-α increases the production of proinflammatory cytokines such as interleukin (IL)-1β and IL-18 in synovial cells [20]. In contrast, IFN-β inhibits expression of IL-1β and tumor necrosis factor (TNF) in peripheral blood mononuclear cells (PBMCs) [21]. Moreover, IFN-β-treated collagen-induced arthritis (CIA) mice exhibited relieved arthritis, suggesting that IFN-β exerts potential therapeutic effects [22]. Beyond expectation, randomized clinical research on the treatment of RA using recombinant IFN-β negated the therapeutic effects of IFN-β in RA [23]. Thus, the effects of IFNs and their response genes and pathways in RA deserve further investigation.

In the past decade, the evolution of high-throughput technologies have made it possible to detect the expression of tens of thousands of genes in one sample at the same time, giving us novel insights into the pathogenesis of RA [24]. Nevertheless, the technologies and analytic tools of single-cell RNA (scRNA) sequencing are rapidly expanding and maturing [25, 26]. Researchers are able to understand the mechanisms of RA in parallel to the dimensions of cell and gene expression with the development of scRNA sequencing [26, 27]. In the present study, we comprehensively analyzed the discrepancies between RA and healthy controls in the terms of both scRNA transcriptomics and conventional RNA transcriptomics of PBMCs. Alterations in type I IFN and IFNs and their response genes and pathways, as well as the resulting potential impacts on the function of immune cells are discussed in detail.

## RESULTS

### Four primary immune cell classes were identified by scRNA transcriptome analysis

**Figure 1:**
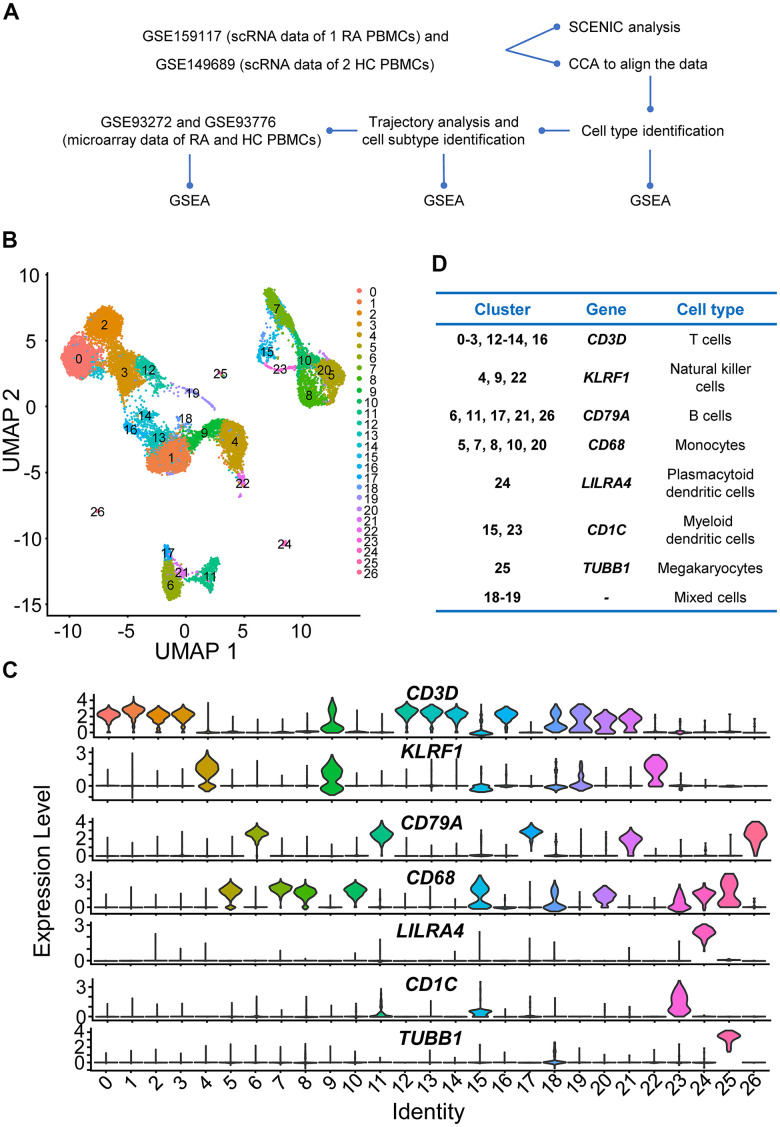
**Study design and preliminary analysis.** (**A**) Workflow of scRNA sequencing and microarray data analysis. Step 1: We first downloaded individual rheumatoid arthritis (RA) and healthy control (HC) peripheral blood mononuclear cell (PBMC) scRNA sequencing data (GSE159117 and GSE149689) from the Gene Expression Omnibus (GEO) database (https://www.ncbi.nlm.nih.gov/geo/). To eliminate the potential batch effects, canonical correlation analysis (CCA) was performed to integrate the two datasets. Afterwards, four main immune cell types (T cells, B cells, NK cells and monocytes) in PBMCs were identified. Then, we explored the up- and downregulated genes and gene sets using differential gene expression analysis and gene set enrichment analysis (GSEA). Subsequently, four main immune cell subtypes were identified using Monocle2, and up- and downregulated interferon (IFN)-related genes and gene sets in different immune cell subtypes were also identified. Step 2: Key transcription factors were identified, and gene regulatory networks (GRNs) were constructed using Single-Cell rEgulatory Network Inference and Clustering (SCENIC) analysis. Step 3: We downloaded microarray datasets including multiple RA and HC PBMC samples and explored the up- and downregulated IFN-related genes and gene sets. (**B**) Two-dimensional uniform manifold approximation and projection (UMAP) visualization of cell clusters. Cells were colored by clusters. (**C**) Violin plots of selected marker genes to identify cell classifications and their expression levels in each cell cluster. (**D**) The table of correspondence between cell clusters and cell types.

To comprehensively explore the disorders in PBMCs from RA, scRNA sequencing data were preprocessed, and batch effects were removed (Supplementary Figure 1A–1D). All cells were grouped to produce 27 clusters first, and all cells were visualized using the principal component analysis (PCA)-based uniform manifold approximation and projection (UMAP) method (Figure 1B). Highly expressed marker genes in each cluster were calculated, and violin plots were used to illustrate the expression of several widely used marker genes in each cluster (Figure 1C).

In the present study, NK cells, monocytes, and T and B cells were identified (Figure 1D), and these cells were conserved for further analysis. Although plasmacytoid DCs (pDCs) and myeloid DCs (mDCs) were also distinguished (Figure 1D), the number of cells was too small to analyze (466 mDCs, 72 pDCs, and 42 megakaryocytes), so those cells were filtered out in subsequent experiments. Moreover, the marker genes of cluster 18 and cluster 19 did not support them as any common or specific cell types in PBMCs, so they were considered as mixed cells (Figure 1D). Considering that clusters 18 and 19 had few cells (the number of all cells in the two clusters was no more than 300), these two clusters were also abandoned.

### Altered IFN-stimulated signaling pathways and genes in NK cells, monocytes, T cells, and B cells from RA PBMCs

**Figure 2:**
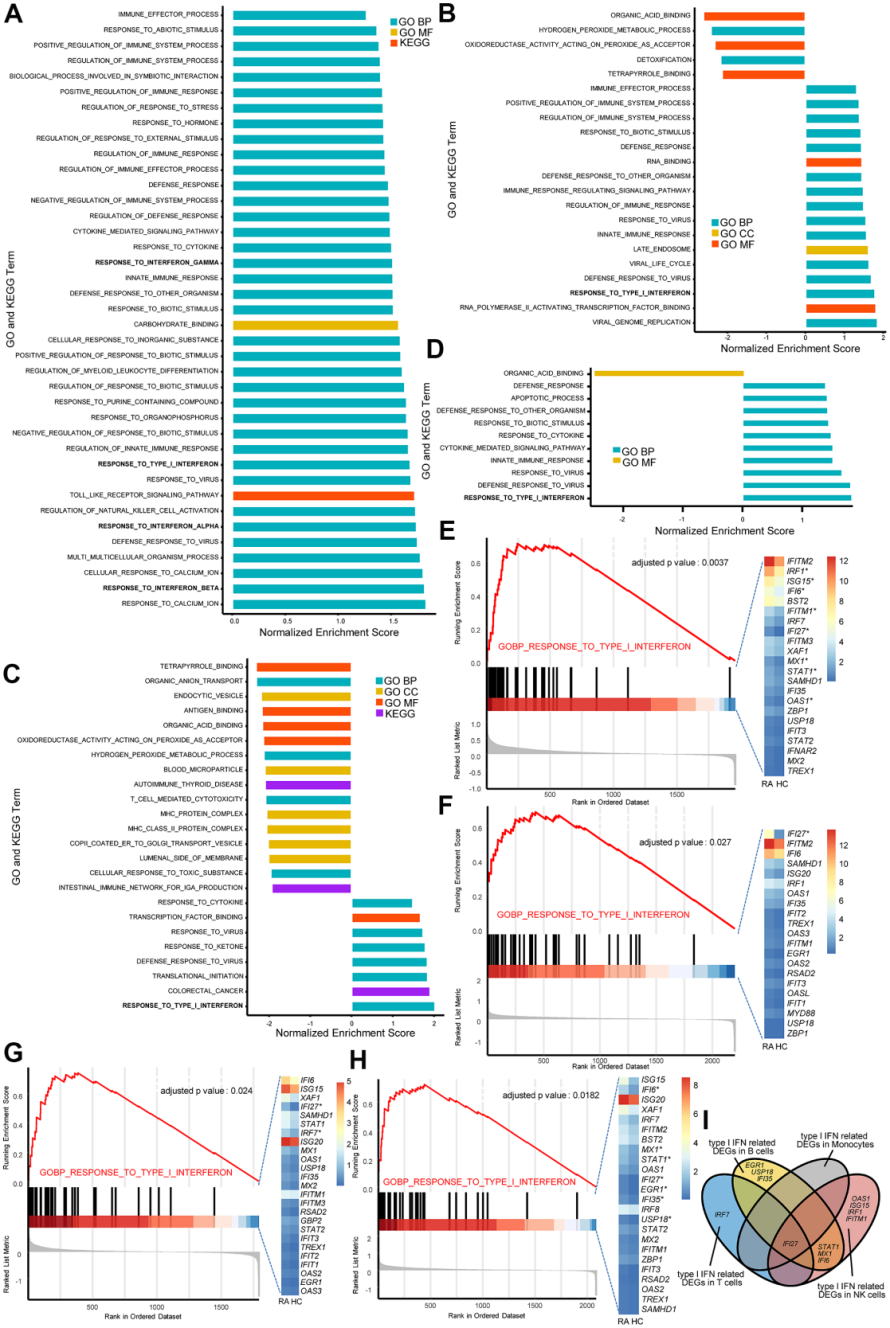
**The type I interferon (IFN) signaling pathway is activated in natural killer (NK) cells, monocytes, and T and B cells in rheumatoid arthritis (RA).** All GSEA bar plots of NK cells (**A**), monocytes (**B**), T cells (**C**) and B cells (**D**) detected by single-cell RNA (scRNA) sequencing data. GSEA plots and Het maps showing the activated type I IFN signaling pathway and genes in NK cells (**E**), monocytes (**F**), T cells (**G**) and B cells (**H**) detected by scRNA sequencing data. Upregulated genes in RA are marked with an asterisk (*). All upregulated genes satisfied log2 (fold change)>0.25 and adjusted p-value<0.05. (**I**) Venn diagram of upregulated type I IFN-stimulated genes in each immune cell type.

Gene set enrichment analysis (GSEA) of NK cells, monocytes, T cells, and B cells was performed to reveal the differences between RA and healthy controls (HCs), and the GSEA results indicated the widespread dysregulation of immune cells in peripheral blood from the RA patient (Figure 2A–2D). Among all significant gene sets, it was not difficult to observe that the Gene Ontology (GO) biological process (BP) pathway named “RESPONSE_TO_TYPE_I_INTERFERON” was upregulated in NK cells, monocytes, T and B cells from RA sample (Figure 2E–2H). Moreover, three other gene sets named “RESPONSE_TO_INTERFERON_ALPHA”, “RESPONSE_TO_INTERFERON_BETA”, and “RESPONSE_TO_INTERFERON_GAMMA” were also activated in NK cells from the RA patient (Figure 2A).

The above results indicate that IFN-α-, and IFN-β-stimulated genes are comprehensively activated in RA peripheral blood. IFN-γ-stimulated genes are also upregulated in RA NK cells. Moreover, there were differentially upregulated IFN-stimulated genes in NK cells, monocytes, T cells, and B cells from RA (Figure 2I), indicating that there were different alterations influenced by IFN in distinct immune cell types. Therefore, we further analyzed the alterations of IFN-α-, IFN-β-, and IFN-γ-stimulated genes in NK cells, monocytes, T cells, and B cells from the RA patient.

### Alterations and influence of IFN stimulated pathways in RA NK cells

**Figure 3:**
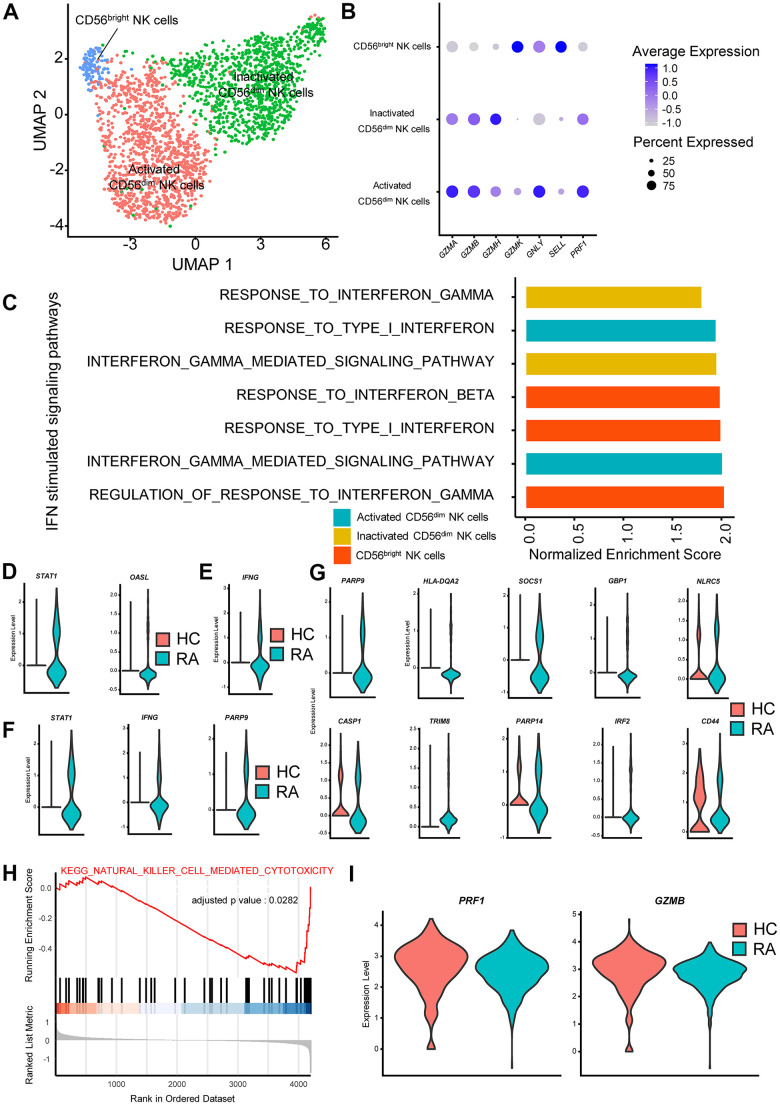
**Interferon (IFN)-stimulated pathways promote cytokine secretion and inhibit cytotoxicity in rheumatoid arthritis (RA) natural killer (NK) cells.** (**A**) Two-dimensional uniform manifold approximation and projection (UMAP) visualization of reclustered NK cells. Three NK cell clusters (activated CD56^dim^ NK cells, inactivated CD56^dim^ NK cells and CD56^bright^ NK cells) were identified. (**B**) Dot plot illustrating the expression levels of several marker genes in three NK cell subtypes. (**C**) Bar plots of selected gene set enrichment analysis (GSEA) results indicated altered IFN signaling pathways in three NK cell subtypes. (**D**) Violin plots of significantly upregulated type I IFN-stimulated genes in RA-activated CD56^dim^ NK cells (*STAT1*) and in RA CD56^bright^ NK cells (*OASL*). (**E**) Violin plot of significantly upregulated IFN-γ-stimulated genes in RA CD56^bright^ NK cells. (**F**) Violin plots of significantly upregulated IFN-γ-stimulated genes in RA-activated CD56^dim^ NK cells. (**G**) Violin plots of significantly upregulated IFN-γ-stimulated genes in RA-inactivated CD56^dim^ NK cells. (**H**) GSEA plot of the “KEGG_NATURAL_KILLER_CELL_MEDIATED_CYTOTOXICITY” pathway in RA inactivated CD56^dim^ NK cells. (**I**) Violin plots of significantly upregulated cytotoxic effector genes in RA activated CD56^dim^ NK cells. All upregulated genes satisfied log2 (fold change)>0.25 and adjusted p-value<0.05.

To better explore the impacts of IFN-stimulated genes and signaling pathways in NKs from RA, we reclustered NK cells and divided them into three subclusters: activated CD56^dim^ NK cells, inactivated CD56^dim^ NK cells and CD56^bright^ NK cells (Figure 3A, 3B). We then examined the transcriptional changes associated with RA in these three NK subclusters using GSEA. The GSEA results showed that IFN-γ stimulated signaling pathways in the three NK subclusters were all activated (Figure 3C). Moreover, type I IFN stimulated signaling pathways were activated in activated CD56^bright^ NK cells and activated CD56^dim^ NK cells (Figure 3C). We also calculated differentially expressed genes (DEGs) in the three NK cell subclusters between the RA patient and HCs, and the results showed that some type I IFN- and IFN-γ-stimulated genes, such as *STAT1* and *SOCS1*, were significantly upregulated in NK cell subclusters (Figure 3D–3G). The above results all indicate that IFNs are partially implicated in NK cell regulation, which eventually causes abnormal changes in the immune environment in peripheral blood. In addition, it is interesting that CD56^dim^ NK cells might produce more IFN-γ according to the results of differential gene expression analysis (Figure 3F), even though CD56^dim^ NK cells primarily exert cytotoxic effects in PBMCs.

We also examined functional changes in NK subclusters in RA, and the inactivated CD56^dim^ NK cells in RA exhibited lower activity in the Kyoto Encyclopedia of Genes and Genomes (KEGG) pathway of “KEGG_NATURAL_KILLER_CELL_MEDIATED_CYTOTOXICITY” (Figure 3H). In activated CD56^dim^ NK cells, expression of the cytotoxic effector molecules *PRF1* and *GZMB* was also significantly depressed (Figure 3I). These results indicate that the cytotoxic capacity of CD56^dim^ NK cells is reduced in RA. Importantly, it is reasonable to believe that those functional changes in RA NK cells are partly associated with the activation of IFN-stimulated genes and pathways based on previous studies [28].

### Alterations and influence of IFN-stimulated pathways in RA monocytes

**Figure 4:**
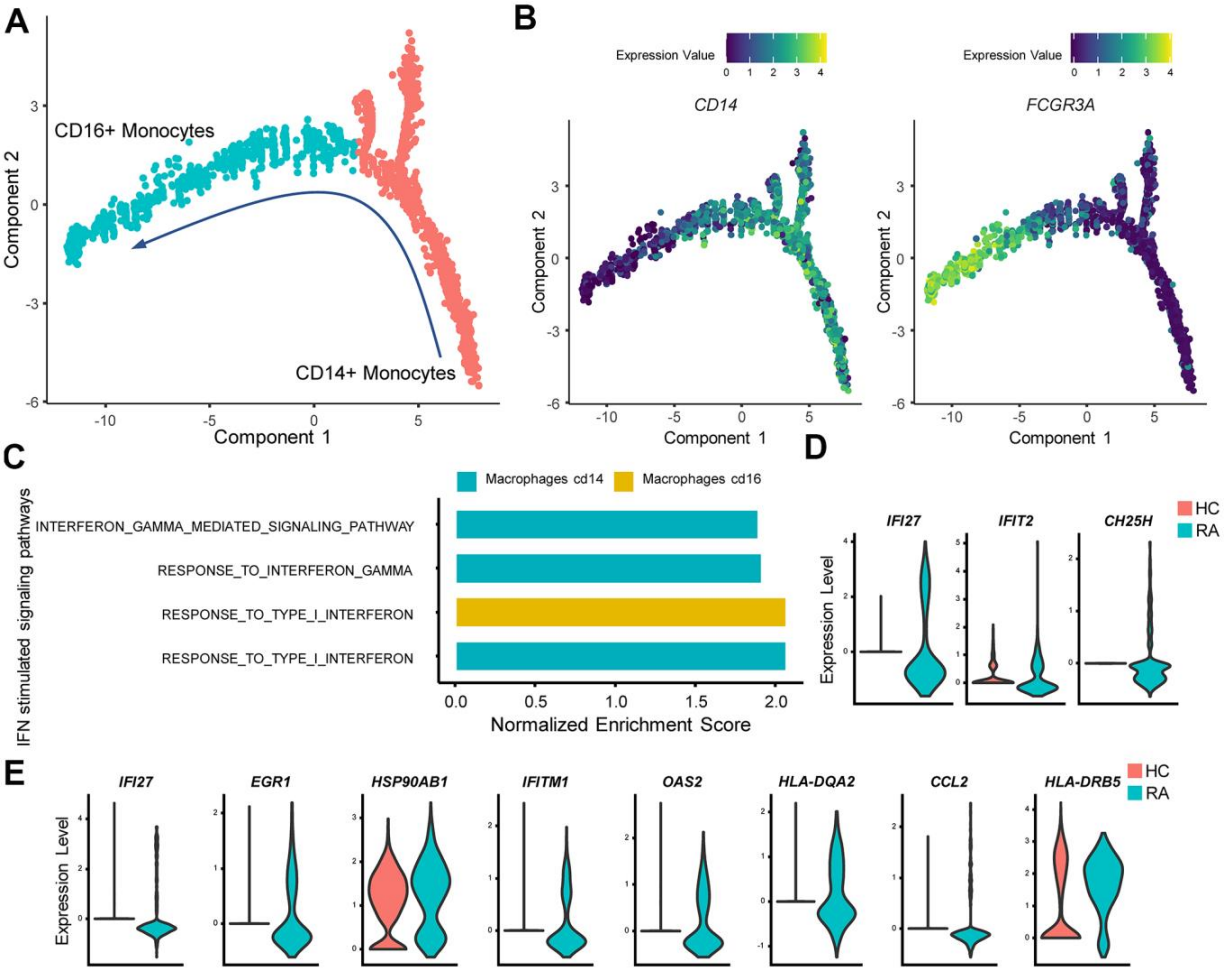
**IFN promotes rheumatoid arthritis (RA) monocyte inflammatory responses.** (**A**) Red and cyan points indicate CD14^+^ monocytes and CD16^+^ monocytes, respectively. The direction of arrows indicates the direction of the pseudotime. (**B**) Trajectory plots of monocytes indicating the expression levels of CD14 and FCGR3A (CD16a) in monocytes. (**C**) Bar plots of selected gene set enrichment analysis (GSEA) results indicate activated IFN signaling pathways in CD14^+^ and CD16^+^ monocytes. (**D**) Violin plots of significantly upregulated type I IFN-stimulated genes (*IFI27, IFIT2*) and upregulated proinflammatory gene (*CH25H*) in RA CD16^+^ monocytes. (**E**) Violin plots of significantly upregulated IFN-γ-stimulated genes (*IFI27, EGR1, HSP90AB1, IFITM1, OAS2, HLA-DQA2, CCL2*) and upregulated proinflammatory genes (*HLA-DRB5*) in RA CD14^+^ monocytes. All upregulated genes satisfied log2 (fold change)>0.25 and adjusted p value<0.05.

In PBMCs, monocytes are commonly divided into CD14^++^CD16^−^ (classical), CD14^++^CD16^+^ (intermediate), and CD14^−^CD16^++^ (nonclassical) populations according to their developmental processes [29]. Here, we followed a similar classification and monocytes were split into CD14^+^ and CD16^+^ monocytes using single-cell trajectory analysis (Figure 4A, 4B). We then analyzed the activity of IFN-stimulated pathways by performing GSEA. CD14^+^ and CD16^+^ monocytes were all activated by type I IFN (Figure 4C, 4D), and CD14^+^ monocytes were activated by IFN-γ simultaneously (Figure 4C, 4E).

CD14^+^ monocytes are more inclined to migrate to local tissues with high levels of *CD62L*, and chemokines (*CXCR2*, *CCR2*), and CD14^+^ monocytes are also considered osteoclast precursors [29–31]. In our study, high HLA-DRB5 expression was also observed in CD14^+^ monocytes from the RA patient (Figure 4E), indicating an inflamed and erosive osteoclast phenotype in RA joint cavities. In contrast, CD16^+^ monocytes primarily secreted inflammatory factors in PBMCs [5]. We also found that CD16^+^ monocytes from RA patients expressed significantly more *CH25H* (Figure 4D), which could promote the inflammatory response in RA peripheral blood [32]. We propose that these inflammatory changes are caused by IFN’s influence according to prior studies [33].

### Alterations and influence of IFN stimulated pathways in RA CD4^+^ and CD8^+^ T cells

**Figure 5:**
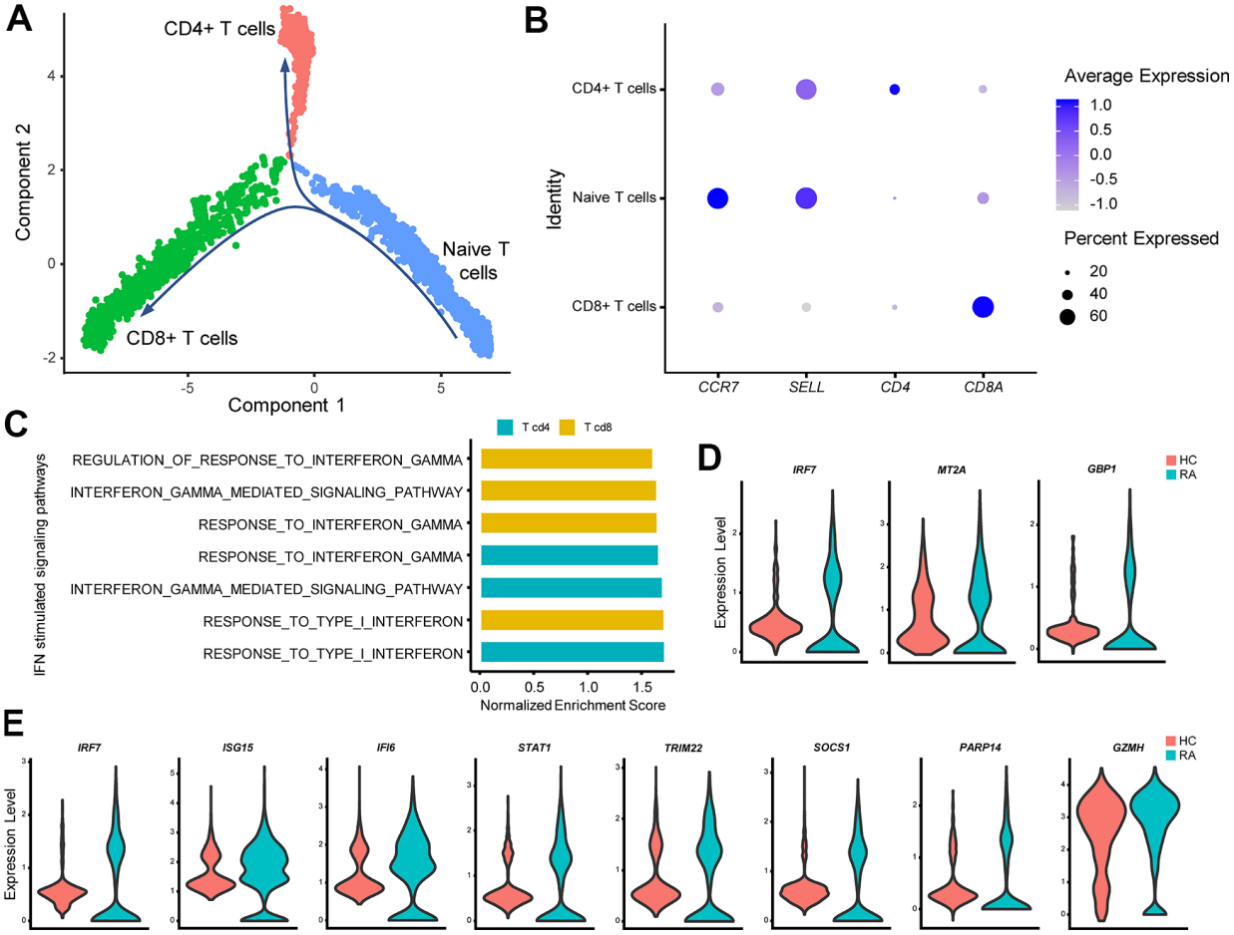
**IFN promotes rheumatoid arthritis (RA) CD4^+^ Th1 polarization and increases RA CD8+ T cell cytotoxicity.** (**A**) Trajectory plots of T cells from RA and healthy control individuals (HC). The direction of arrows indicates the direction of the pseudotime. (**B**) Dot plot illustrating the expression levels of several marker genes in three T cell subtypes. (**C**) Bar plots of selected gene set enrichment analysis (GSEA) results indicate activated IFN signaling pathways in CD4^+^ and CD8^+^ T cells. (**D**) Violin plots of significantly upregulated IFN-γ-stimulated genes in RA CD4^+^ T cells. (**E**) Violin plots of significantly upregulated IFN-γ-stimulated genes (*IRF7*, *ISG15*, *IFI6*, *STAT1*, *TRIM22*, *SOCS1*, *PARP14*) and the cytotoxic effector gene (*GZMH*) in RA CD8^+^ T cells. All upregulated genes satisfied log2 (fold change)>0.25 and adjusted p-value<0.05.

The heterogeneous nature of T cells makes investigation of T cells complex. For convenience, T cells were split into three subgroups: CD4^+^ T cells, CD8^+^ T cells, and naïve T cells via single-cell trajectory analysis (Figure 5A, 5B). GSEA indicated that type I IFN- and IFN-γ-stimulated pathways were activated in CD4^+^ T cells and CD8^+^ T cells but not in naïve T cells (Figure 5C). Meanwhile, expression of IFN-stimulated genes such as *IRF7* was significantly increased in CD4^+^ T cells and CD8^+^ T cells from the RA patient (Figure 5D, 5E). In addition, levels of GZMH were increased in RA CD8^+^ T cells (Figure 5E), which might also be caused by type I IFN activation [34].

### Alterations and influence of IFN stimulated pathways in RA naïve B cells and plasma cells

**Figure 6:**
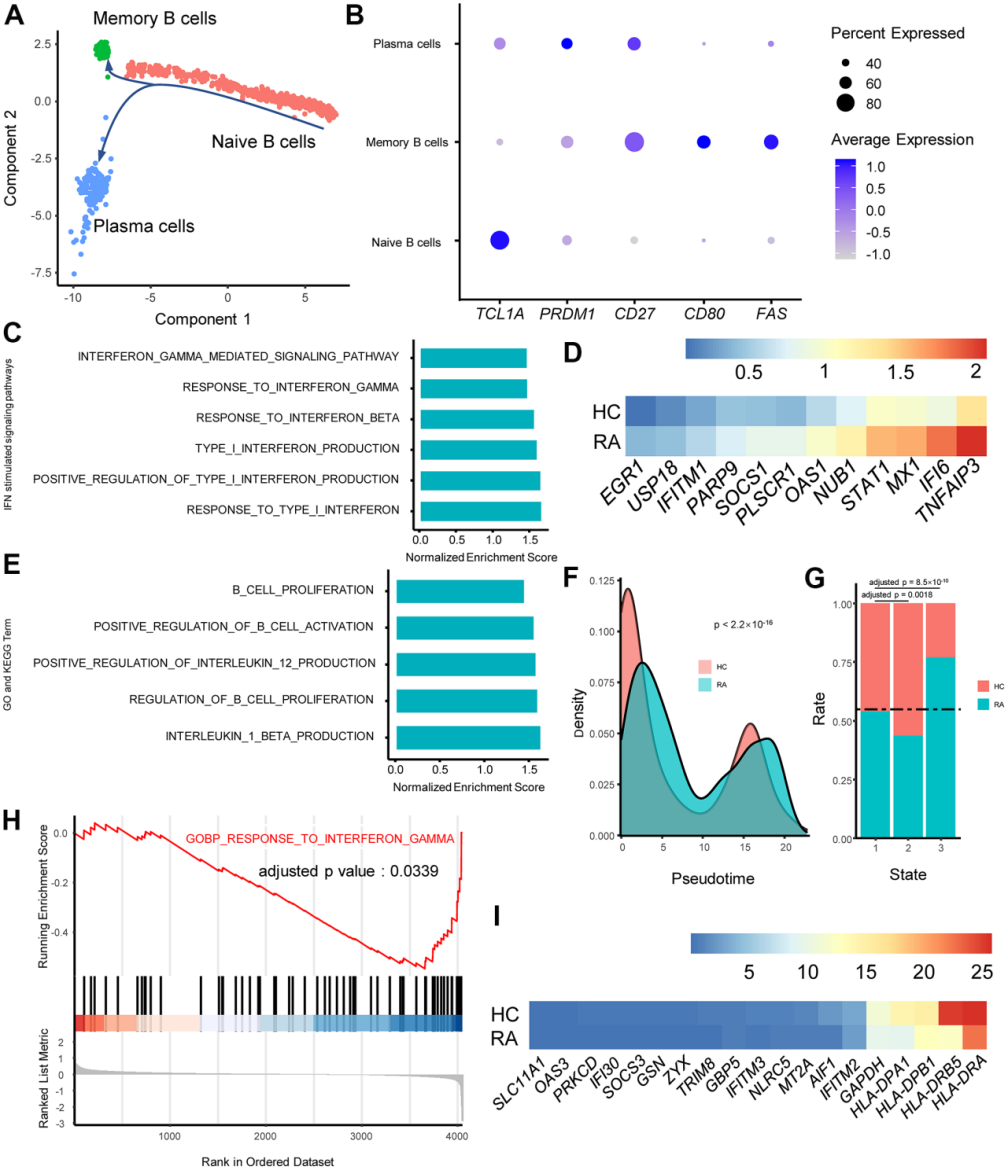
**IFN alters rheumatoid arthritis (RA) B cell proliferation and activation and class switching in RA plasma cells.** (**A**) Trajectory plots of T cells from RA and healthy control individuals (HC). The direction of arrows indicates the direction of pseudotime. (**B**) Dot plot illustrating the expression levels of several marker genes in three T cell subtypes. (**C**) Bar plots of selected gene set enrichment analysis (GSEA) results indicate activated IFN signaling pathways in RA naïve B cells. (**D**) Heat map of upregulated type I IFN stimulated genes in RA naïve B cells. Genes are ordered according to their expression levels in RA naïve B cells. (**E**) GSEA results indicate activated B cell functions in RA naïve B cells. (**F**) Probability density plot of B cell pseudotimes in RA and HC. Pseudotimes between the RA patients and HCs were compared using the Mann-Whitney U test. (**G**) Stacked bar plot of B cell subtype distribution in RA and HC. The horizontal dotted line indicates the overall B cell proportions in RA and HC. The proportions of memory B cells and plasma cells in the RA patient and HCs were compared to the proportions of naïve B cells, and comparisons were performed using Fisher’s exact test. (**H**) GSEA plot of the “GOBP_RESPONSE_TO_INTERFERON_GAMMA” pathway in RA plasma cells. (**I**) Heat map of downregulated IFN-γ-stimulated genes in RA plasma cells. Genes are ordered according to their expression levels in HC naïve B cells.

For B cells, cell trajectory analysis was performed, and the track plot of B cell trajectories is shown in Figure 6A. Three B cell clusters included naïve B cells, plasma cells, and memory B cells according to their marker genes (Figure 6B). GSEA results showed that type I IFN and IFN-γ primarily affected naïve B cells (Figure 6C), and upregulated type I IFN-stimulated genes were also observed in RA naïve B cells (Figure 6D). Moreover, GSEA results indicated that naïve B cells were one of the sources of type I IFN (Figure 6C).

Remarkably, the GSEA results showed that naïve B cells from RA were activated and tended to proliferate faster (Figure 6E). *EGR1* has been demonstrated to promote B cell differentiation into plasma cells and support antibody secretion, and higher expression of *EGR1* was identified in naïve B cells from RA (Figure 6D) [35]. The pseudotimes of B cells were also significantly different between the two groups, and RA plasma cells exhibited older pseudotimes (Figure 6F), indicating that RA B cells are primarily concentrated at the end of the differentiation trajectory. Moreover, the proportions of three B cell types in RA and HC were compared, and the results revealed that the number of plasma cells in RA was increased with the same proportion of naïve B cells (Figure 6G). In addition, GSEA results demonstrated that naïve B cells from RA tended to produce more of the proinflammatory factors IL-1β and IL-12 (Figure 6E).

Interestingly, plasma cells in RA exhibited lower activity in the “RESPONSE_TO_INTERFERON_GAMMA” pathway (Figure 6H), and the expression of IFN-γ-stimulated genes, such as IFITM3 and IFI30, were downregulated in RA plasma cells (Figure 6I). In accordance with previous studies, this might affect antibody class switching in RA plasma cells [36].

### Single-cell regulatory network inference and clustering (SCENIC) analysis revealed the chief transcription factors and gene regulatory networks (GRNs) in RA PBMCs

**Figure 7:**
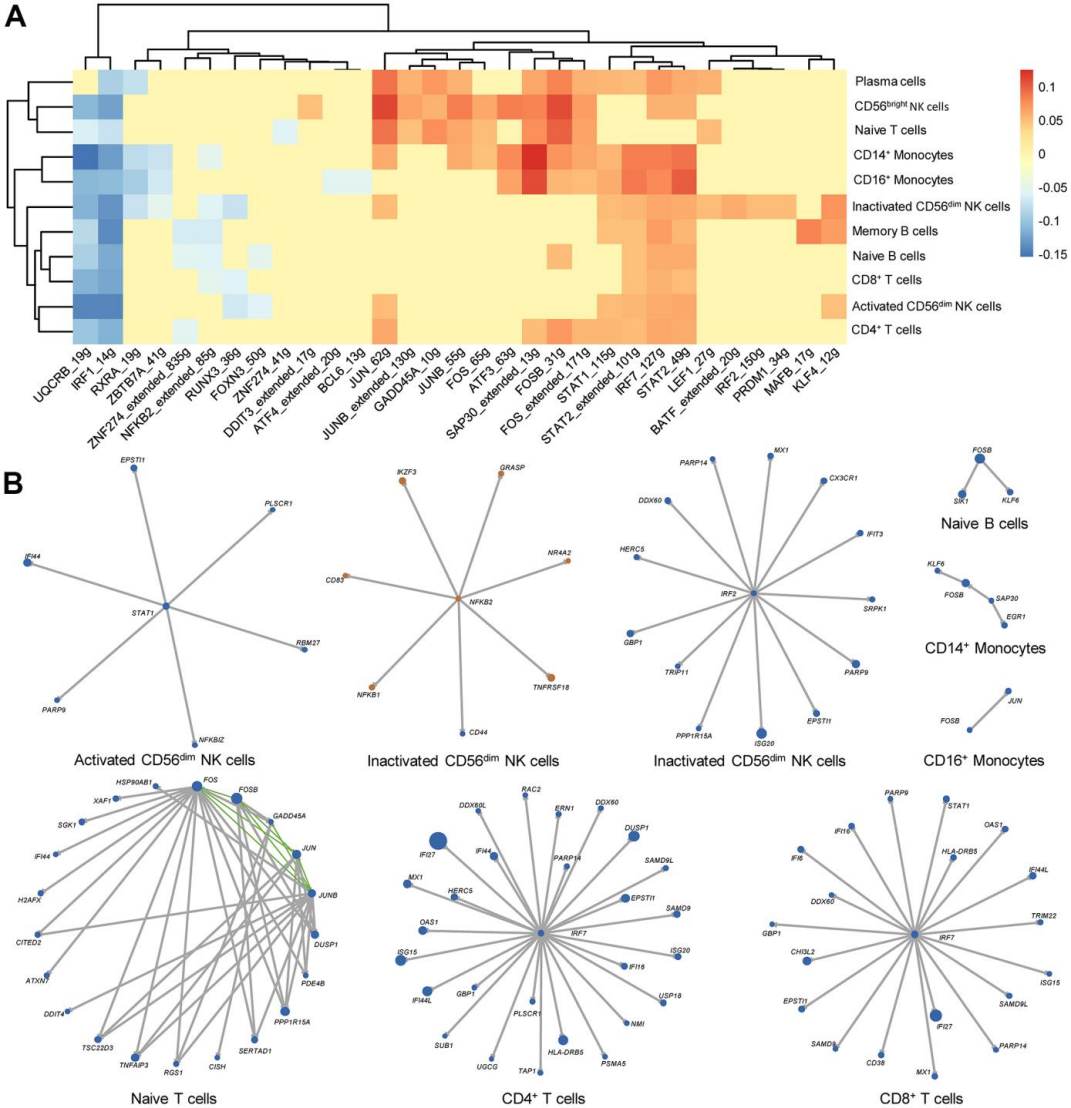
**Key transcription factors and gene regulatory networks (GRNs) in each immune cell type from rheumatoid arthritis (RA) peripheral blood.** (**A**) Heat map of differential regulons in each immune cell type. The heat map color indicates the difference in AUC values in RA patients and HCs, and only significantly different regulons are shown in the heat map. Red and blue indicated that the regulon is upregulated and downregulated in RA, respectively. (**B**) GRNs in each immune cell type. Blue and orange dots represent upregulated and downregulated genes in RA, respectively. The size of the dots corresponds to the absolute value of fold changes. Gray lines and green lines represent unidirectional and bidirectional regulatory relationships, respectively.

The effects of IFNs on immune cells are complex. Here, we focused on the regulation of downstream transcription factors and their target genes by IFNs. We first calculated and compared the AUC values of the regulon between RA and HC, and significantly differential regulons are illustrated in Figure 7A. Regulons of IRF7_127g, STAT1_115g, STAT2_49g and STAT2_extended_101g were significantly upregulated in most cell types. Instead, the regulons of IRF1_14g and UQCRB_19g were significantly decreased in most cell types (Figure 7A). Subsequently, we constructed GRNs in different immune cell types. Many IFN-stimulated genes were included in the GRNs (Figure 7B), and different cell types exhibited diverse IFN-stimulated genes, indicating different and fine regulation of gene expression in distinct immune cells.

### External datasets validated the activation of interferon-simulated pathways and genes in peripheral blood from RA patients

Considering that the above results came from a single RA patient, we used external transcriptomic data from multiple individuals to explore the expression of IFN-stimulated genes and pathways in RA PBMCs. We first analyzed a microarray dataset including 232 RA PBMC samples and 43 HC PBMC samples, and the GSEA results demonstrated that the “GOBP_RESPONSE_TO_TYPE_I_INTERFERON” and “GOBP_RESPONSE_TO_INTERFERON_GAMMA” pathways were activated in RA (Supplementary Figure 2A, 2B), and related genes were also upregulated in RA (Supplementary Figure 2C, 2D). Furthermore, we investigated alterations in IFN-stimulated pathways and genes in different cell types in RA PBMCs. The results indicated that the effects of type I IFN were primarily concentrated in CD14^+^ monocytes (Supplementary Figure 3), while the effects of IFN-γ were primarily concentrated in monocytes, effector memory CD4^+^ T cells and NK cells (Supplementary Figure 3). In addition, the GSEA results also suggested that NK cells in RA produced more IFN-γ (Supplementary Figure 3), which is consistent with the results of scRNA sequencing analysis.

## DISCUSSION

IFNs are a series of natural cytokines that exert pivotal immunomodulatory activities [37–39]. Both type I and II IFN are presumed to be the bridge between innate immune and adaptive immune responses due to their functions in enhanced antigen presentation. From this point, the intensities of type I and II IFN signaling pathways are crucial for the balance between self-reactivity and autoimmunity, illustrating that IFNs play important roles in autoimmune diseases.

Type I IFN can be produced by many immune cell types, including DCs, monocytes, and macrophages [13]. T cells and NK cells are the major producers of IFN-γ [40]. Type I and II IFN can also act on immune cells in an autocrine fashion. The influences of IFN signaling on immune cells are comprehensive and involve multiple signaling pathways. The type I IFN receptor (IFNAR) activates its downstream target kinases, including JAK1 and TYK2, which promotes phosphorylation, dimerization, and nuclear translocation of STAT1 and STAT2 [41]. Finally, the expression of a series of genes called IFN-stimulated genes is facilitated. IFN-γ also regulates gene expression by activating the JAK-STAT1 signaling pathway and other factors, including the AP-1, STAT3, STAT5, the MAP kinase signaling pathway, the PI3K signaling pathway and the NF-κB signaling pathway [42].

NK cells, T and B cells and monocytes are the crucial proportions of PBMCs, which are also involved in innate and acquired immune responses and are related to a series of autoimmune diseases. GSEA is a popular and useful tool to identify specific up- or downregulated pathways. In the present study, we determined that IFN-related signaling pathways were activated in PBMCs from RA microarray data using GSEA and GO and KEGG pathways from the Molecular Signatures Database (MSigDB). Then we focused on the abnormal expression of type I and II IFN-related genes and gene sets, and consequent alterations in NK cells, monocytes, and T and B cells from RA peripheral blood.

CD56^dim^ NK cells exert roles in the immune response primarily through there cytotoxic effects on abnormally activated T cells and macrophages, and CD56^bright^ NK cells primarily regulate the immune response by producing cytokines [43]. Aberrant alterations of NK cells partially contribute to the progression of RA. It has been reported that there are increased CD56^bright^ NK cells in inflamed synovial joints, which also express more IFN-γ than NK cells in the peripheral blood [44]. Previous research also demonstrated that NK cell activity was impaired in RA [45].

NK cell activation relies on the stimulation of type I IFN [28]. In our study, we found that both type I IFN- and IFN-γ-stimulated signaling pathways were activated in activated CD56^dim^ NK cells and CD56^bright^ NK cells. Notably, in agreement with prior studies, our study also suggested decreased cytotoxic effects of RA activated and inactivated CD56^dim^ NK cells [45].

It has been reported that NK cells stimulated with IFN-γ exhibit increased phosphorylation of STAT1 or STAT4, and different activation of STATs leads to distinct NK cell phenotypes [28]. Specifically, STAT1 phosphorylation promotes the cytotoxic activity of NK cells, while STAT4 phosphorylation promotes NK cell cytokine secretion [46, 47]. Although a higher level of *STAT1* in RA-activated CD56^dim^ was detected, we speculated that the impaired NK cell activity in RA CD56^dim^ NK cells was related to IFN-γ stimulation and downstream STAT4 phosphorylation.

Aberrant activation of monocytes also contributes to RA. CD14^+^ monocytes are more inclined to migrate to local tissues including joint cavities [29, 30]. A large number of peripheral CD14^+^ monocytes differentiate into osteoclasts in RA, ultimately leading to bone erosion. Herein, our study indicated that CD14^+^ monocytes from RA exhibited increased HLA-DRB5 levels and IFN-γ signaling pathway activation. CD14^+^CD16^+^HLA-DR^+^ monocytes secrete high levels of TNF, which indicates that HLA-DR^+^ monocytes exhibit a potential proinflammatory phenotype [48]. IFN-γ has been considered an effective inducer of HLA-DR expression on synovial monocytes by *in vitro* cell experiments [5]. Taken together with the above results, we speculate that higher expression of HLA-DR in CD14^+^ monocytes indicates stronger immune responses in RA peripheral blood.

CD16^+^ monocytes in RA also exert momentous effects on RA by producing proinflammatory cytokines, such as IL-1β, IL-6, and TNF-α [5]. In our study, we found that RA CD16^+^ monocytes expressed more *CH25H* than HCs, accompanied by activation of the type I IFN signaling pathway. *CH25H* is an enzyme that catalyzes the formation of 25-hydroxycholesterol (25HC) from cholesterol, and it has been demonstrated that *CH25H* acts as an inflammatory signaling amplifier in macrophages [32, 49]. Moreover, individuals with higher *CH25H* expression in synovial membranes are more likely to develop RA [50]. Previous research has demonstrated that *CH25H* is an IFN-stimulated gene and that expression of *CH25H* is induced by IFN-α [33]. Thus, we hypothesized that type I IFN promotes the expression of *CH25H* in RA CD16^+^ monocytes, which aggravated the inflammatory response in RA CD16^+^ monocytes. In addition, although expression of *CH25H* represents an inflammatory signal, the relationship between *CH25H* and RA needs further investigation because there are few relevant studies.

Consistently, CD4^+^ T cells occupy a core status in RA [51]. CD4^+^ T cells contain a CD4^+^ T cell subtype mixture of Th1, Th2, Th17, and regulatory T cells (Tregs). Among them, enhanced Th1 and Th17 activities, as well as elevated IFN-γ and IL-17, promote inflammatory responses both in synovial membranes and in PBMCs [52]. Our study demonstrated that the IFN-γ stimulated signaling pathway was activated in RA CD4^+^ T cells, indicating that it enhanced CD4^+^ Th1 polarization in RA. These results are consistent with previous studies [52]. CD8^+^ T cells also function in RA, but their precise roles in RA pathogenesis are still unclear [53]. Some animal studies have demonstrated that CD8^+^ T cells have proinflammatory effects via cytotoxicity [54]. However, other studies have indicated that CD8^+^ T cells play a regulatory role in inflamed joints [55]. In the present study, we found that *GZMH*, a cytotoxic gene, was upregulated in RA CD8^+^ T cells, supporting the view that the cytotoxicity of CD8^+^ T cells is enhanced in RA. It is also known that IFN-γ can enhance the cytotoxicity of CD8^+^ T cells. Therefore, we hypothesized that the cytotoxicity of CD8^+^ T cells was elevated and was associated with the activation of IFN-γ-stimulated pathways [34].

In RA, unnatural B cell activation leads to the production of autoantibodies, including anti-cyclic citrullinated peptide (anti-CCP) and rheumatoid factor (RF). Unsurprisingly, we found that naïve B cells in RA exhibited stronger proliferation and activation, accompanied by the activation of activation of type I IFN. It has been previously demonstrated that type I IFN boosts B cell proliferation and differentiation to plasma cells [56]. *EGR1*, a type I IFN-stimulated gene, was also upregulated in RA naïve B cells [35]. Thus, we hypothesized that the activation of type I IFN partially enhanced B cell differentiation into plasma cells.

Activated B cells also regulate immune responses by secreting cytokines [36]. Both proinflammatory cytokines and anti-inflammatory cytokines can be produced by B cells, depending on the stimulus [57]. It has been reported that B cells from the autoimmune disease multiple sclerosis produce less of the anti-inflammatory cytokine IL-10 [58]. Here, our study indicated that naïve B cells in RA had the potential to produce the proinflammatory cytokines IL-1β and IL-12, which might aggravate the inflammatory status in RA peripheral blood. Whether the cytokines from RA B cells are related to type I IFN and IFN-γ activation remains to be further investigated.

Inhibition of the IFN-γ signaling pathway in RA plasma cells is another interesting phenomenon observed in the present study. Previous studies have demonstrated that IFN-γ intervenes in plasma cell isotype switch recombination by promoting IgG2a production and inhibiting IgG1 production [59, 60]. Coincidentally, IgG1 and IgG4 are the primary subtypes of anti-CCPs in RA [61, 62]. As a result, we conjectured that the inhibited IFN-γ signaling pathway in plasma cells might be associated with plasma cell isotype switch recombination in RA, which eventually promotes the production of autoantibodies.

Finally, IFN-regulated pathways and genes are complex and numerous. Therefore, we attempted to use SCENIC to evaluate the activity of each transcription factor and their target genes. We found that the activities of the regulons IRF7_127g, STAT1_115g, STAT2_49g, STAT2_extended_101g, IRF1_14g and UQCRB_19g were significantly increased or decreased in most cell types. There were several imbalanced transcription factors in RA, such as *IRF1*, *IRF7*, *STAT1*, and *STAT2.* They could be regulated by IFN, addressing the important IFN effects in RA [63–65]. Furthermore, we constructed GRNs by integrating the results of SCENIC analysis and DEG analysis in each cell type. Our GRNs might not be comprehensive due to the strict calculations of DEGs. Moreover, only a single RA sample with missing clinical characteristics was used in the main analysis of the present study, which limited the generalizability of the findings. Further research is required to explain the significance of these transcription factors in RA. Once their expression and functions are identified, these transcription factors could be used as potential targets in the treatment of RA due to their extensive influence instead of partial alterations in RA.

## CONCLUSIONS

Knowledge of the relationship between IFN and RA is tortuous because IFN participates in multiple regulatory effects on the immune system [66]. Initially, type I IFN was used as a treatment option [67]. Subsequently, the promoting effects of IFN-stimulated signaling pathways on RA have been gradually recognized [68]. The type I IFN signature represents type I IFN response genes and pathways, that are activated in RA patients, which may play a role in the potential development of RA [11]. Meanwhile, contradictory findings indicated the dual effects of IFN-γ on RA, and our findings demonstrated that IFN-γ-stimulated pathways were not always activated in all cell types in RA [69].

In the present study, we comprehensively analyzed the alterations of type I IFN- and IFN-γ-stimulated pathways in RA. Parts of the findings from the transcriptome analysis corroborate previous studies. The present study indicated that IFN signaling pathways are activated in RA patients, in agreement with prior investigations. A previous study showed that CD56^dim^ NK cells from RA patients are impaired, and our study supported that conclusion [45]. Enhanced Th1 activity and imbalanced Th1/Th2 cells in RA were detected very early [51]. Our research also found enhanced IFN-γ signaling pathway in RA CD4^+^ T cells, indicating that Th1 polarization is enhanced in RA CD4^+^ helper T cells. However, the subtypes of CD4^+^ helper T cells are diverse, and the relationship between the Th17/Treg balance and RA has been observed in prior research [51]. How IFN signaling pathways and CD4^+^ helper T cell differentiation influence each other needs to be clearly demonstrated in future studies.

Our study also delivers some novel findings. We linked disease promotion of CD56^bright^ and CD56^dim^ NK cells in RA with activated IFN signaling pathways. Our research also indicated that the activated type I IFN signaling pathway might promote production of the inflammatory signaling amplifier CH25H. In addition, RA patient primary B cells tended to differentiate into plasma cells, and our investigation demonstrated that the type I IFN signaling pathway might be one of the factors causing this differentiation. Our analysis also demonstrated that the IFN-γ signaling pathway in RA was activated, which might influence antibody class switching in RA plasma cells and autoantibody production. Finally, SCENIC was used to identify key transcription factors in different immune cells in RA, and GRNs were also constructed to reveal the mechanism of transcription regulation, which provides directions for future research on IFN signaling pathways in RA.

Although this study comprehensively explored the effects of abnormal type I and II IFN signaling pathways in RA PBMC immune cell subtypes, only one RA scRNA sample was used in our study, and the clinical characteristics of the RA patient were unknown. Thus, we suggest that further studies should focus on IFN-stimulated signaling pathways in RA using multiple samples and corresponding clinical information. Additionally, it is crucial to distinguish different INF signal alterations in distinct cell types to obtain a better cell classification performance, cell sorting is worth using before performing scRNA sequencing. Regardless, it is questionable whether IFN signaling pathways directly and/or indirectly promote the occurrence and development of RA according to previous studies. The relationship between IFN signaling pathways and RA immune cells should be carefully evaluated with a series of *in vitro* and *in vivo* experiments using a combination of novel sequencing technologies and traditional molecular biology techniques.

## MATERIALS AND METHODS

### Acquisition of single-cell sequencing data and microarray data

Both PBMC scRNA sequencing data and mRNA expression microarray data were downloaded from the Gene Expression Omnibus (GEO) database (https://www.ncbi.nlm.nih.gov/geo/). PBMC scRNA sequencing data of one RA patient and two HCs were obtained from GSE159117 and GSE149689, respectively [70, 71]. The mRNA expression data of 232 RA and 43 HC PBMC samples, which were preprocessed by frozen robust multi-array analysis (fRMA) with batch effects corrected, were obtained from GSE93272 [72]. Microarray data preprocessed by fRMA of several immune cell subtypes in PBMCs were obtained from GSE93776 [72]. The design of this research is shown in Figure 1A.

### Data preprocessing and quality control

For scRNA sequencing data, the R package Seurat (version 4.0.0) was used to preprocess the scRNA sequencing data [73–76]. First, cells were included if they met all three of the following parameters: (1) the number of genes in each cell was greater than 500; (2) the total number of molecules in a cell was greater than 1000 and less than 15000; and (3) the mitochondrial gene expression ratio was less than 5% and 15% for GSE159117 and GSE149689, respectively. Two GEO series scRNA data were normalized using “NormalizeData” function, and 3000 highly variable genes were identified using “FindVariableFeatures”. Second, two GEO series were integrated in Seurat using the canonical correlation analysis (CCA) method with the “FindIntegrationAnchors” and “IntegrateData” functions.

Subsequently, the PCA method was performed for dimension reduction after data scaling, and the top 30 principal components were selected to perform the downstream analysis. The UMAP algorithm was used to visualize and explore the data. Cell clusters were identified by the function “FindNeighbors” using the K-nearest neighbors (KNN) algorithm and the function “FindClusters” with a resolution of 1.25.

### Cell class identification

We calculated marker genes to annotate cell clusters to specific immune cell types. The following marker genes were used for cell type annotation: *CD3D*, *KLRF1*, *CD79A*, *CD68*, *LILRA4*, *CD1C*, and *TUBB1* [77–80]. Correspondence between marker genes and cell types is shown in Figure 1C. Considering that the numbers of pDCs, mDCs, and megakaryocytes were too small, they were excluded from all subsequent analyses.

### Single-cell trajectory analysis and cell subtype identification

To estimate the dissimilar functions of the different immune cell types in detail, the four main classes of immune cells (NK cells, monocytes, T cells, and B cells) were further divided according to the general classification criterion and certain marker genes. NK cells identified above were chosen, and reclustering was performed in Seurat using the same pipeline as previously described. NK cells were then subdivided into three clusters, and the following genes were used for NK cell subtype annotation: *PRF1*, *GZMA*, *GZMB*, *GNLY* (activated CD56^dim^ NK cells), *GZMH* (inactivated CD56^dim^ NK cells), and *GZMK* and *SELL* (CD56^bright^ NK cells) [81, 82]. For monocytes, T cells and B cells, the R package monocle (version 2.18.0) was used to perform single-cell trajectory analysis, and pseudotime trajectories were constructed using the DDRTree algorithm [83]. Subsequent genes were used for cell subtype identification: *CD14* (CD14^+^ monocytes), *FCGR3A* (CD16^+^ monocytes), *CCR7*, *SELL* (naïve T cells), *CD4* (CD4^+^ T cells), *CD8A* (CD8^+^ T cells), *TCL1A* (naïve B cells), *PRDM1* (plasma cells), *FAS*, *CD80* and *CD27*(memory B cells) [78, 84–86].

### Differentially expressed gene identification

DEGs between cells from two individuals are important for determining their potential distinct biological functions. To identify DEGs between RA and HC in different immune cell subtypes, we used the FindMarkers function in Seurat to evaluate them and set min.pct = 0.1, logfc.threshold = 0.25, only.pos = FALSE, and only genes with adjusted p-value < 0.05 were retained.

### Gene set enrichment analysis

GSEA was performed using the R package clusterprofiler (version: 3.18.1) [87]. Gene set files were downloaded from http://www.gsea-msigdb.org/gsea/downloads.jsp, and all GO gene sets and KEGG gene sets were used for enrichment analysis. For scRNA sequencing data, genes were ranked using the “FindMarkers” function in Seurat. For microarray data, gene log2 (fold-change) values were calculated using the R package limma (version: 3.46.0), and all genes were decreasingly ranked by their log2 (fold-change) values [88]. The normalized enrichment score (NES) was used to assess the results of gene set enrichment. Pathways with an adjusted p-value<0.05 and |NES|>1 were considered significant. GSEA plots were created using the R package enrichplot (https://yulab-smu.top/biomedical-knowledge-mining-book/, version: 1.10.2). Bar plots were generated using the R package ggpubr (https://CRAN.R-project.org/package=ggpubr, version: 0.4.0).

### Single-cell regulatory network inference and clustering analysis and regulatory network construction

SCENIC is a computational method to infer GRNs from single-cell RNA-seq data [89]. SCENIC analysis was performed according to the official workflow. We used the R package SCENIC (version: 1.2.4) and the GRNboost2 algorithm in the python package arboreto (version: 0.1.5) to assess the gene regulatory relationships in subclasses of PBMCs [90]. Genes that were expressed at either very low levels or in too few cells were removed first; subsequently, we split the targets into positive- and negative-correlated targets by calculating the correlation in R. Gene coexpression networks were then constructed using the GRNboost2 algorithm in Python and SCENIC in R. AUC values of the regulon were calculated to measure the activity of the regulon. A heat map of regulon AUC values in each cell type was illustrated and clustered using R the package pheatmap (version: 1.0.12). Finally, the AUC values of the regulon in each subclass cell were compared using the limma package between the RA patients and HCs. Only a regulon with a logFC≥0.05 and adjusted p-value < 0.05 was considered significantly different between RA and HC groups. Finally, genes belonging to both different regulons between RA and HC and DEGs identified using the FindMarkers function were used for GRN construction.

### Statistical analysis

We compared B cell pseudotime between RA and HC by using Mann-Whitney U test by function “wilcox.test” in R. Differences in B cell subtype proportions were compared using Fisher’s exact test using the function “pairwise.fisher.test” in the R package fmsb (https://CRAN.R-project.org/package=fmsb, version: 0.7.0), and p-values were adjusted using a Benjamini–Hochberg (BH) method. A p-value < 0.05 was considered significant.

### Data availability

Publicly available datasets were analyzed in this study. This data can be found here: https://www.ncbi.nlm.nih.gov/geo/query/acc.cgi?acc=GSE159117; https://www.ncbi.nlm.nih.gov/geo/query/acc.cgi?acc=GSE149689; https://www.ncbi.nlm.nih.gov/geo/query/acc.cgi?acc=GSE93272; https://www.ncbi.nlm.nih.gov/geo/query/acc.cgi?acc=GSE93776.

## Supplementary Material

Supplementary Figures
