# Supplementary material for: A comprehensive transcriptomic analysis of alternate interferon signaling pathways in peripheral blood mononuclear cells in rheumatoid arthritis
Source: Aging (Albany NY). 2021 Aug 25;13(16):20511–33. doi: 10.18632/aging.203432 (PMC8436925; doi:10.18632/aging.203432)
Supplement: Supplementary Figures [file aging-13-203432-s001.pdf]

SUPPLEMENTARY FIGURES

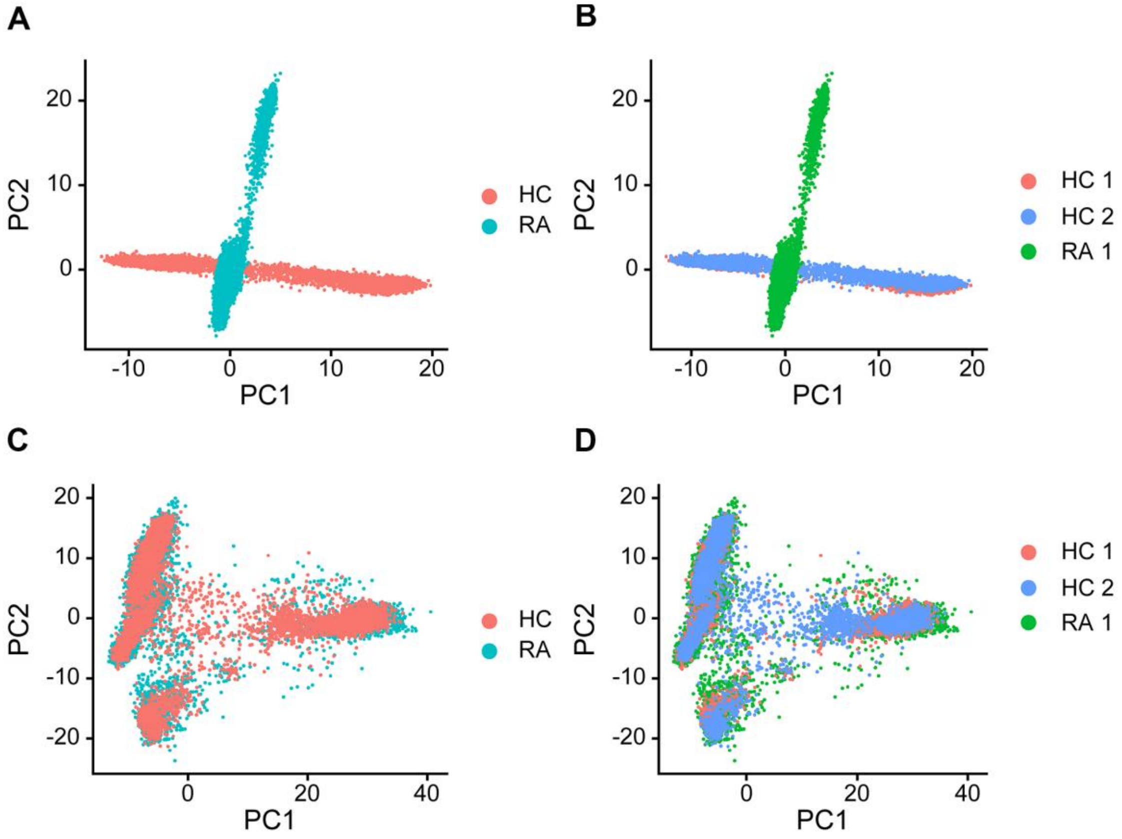

Supplementary Figure 1. Principal component analysis (PCA) visualization suggested that batch effects were removed between rheumatoid arthritis (RA) and healthy control individuals (HC) by canonical correlation analysis (CCA). (**A**) PCA visualization of cells before performing CCA, and cells are colored by batch. (**B**) PCA visualization of cells before performing CCA, and cells are colored by sample. (**C**) PCA visualization of cells after performing CCA, and cells are colored by batch. (**D**) PCA visualization of cells after performing CCA, and cells are colored by sample. PC: principal component.

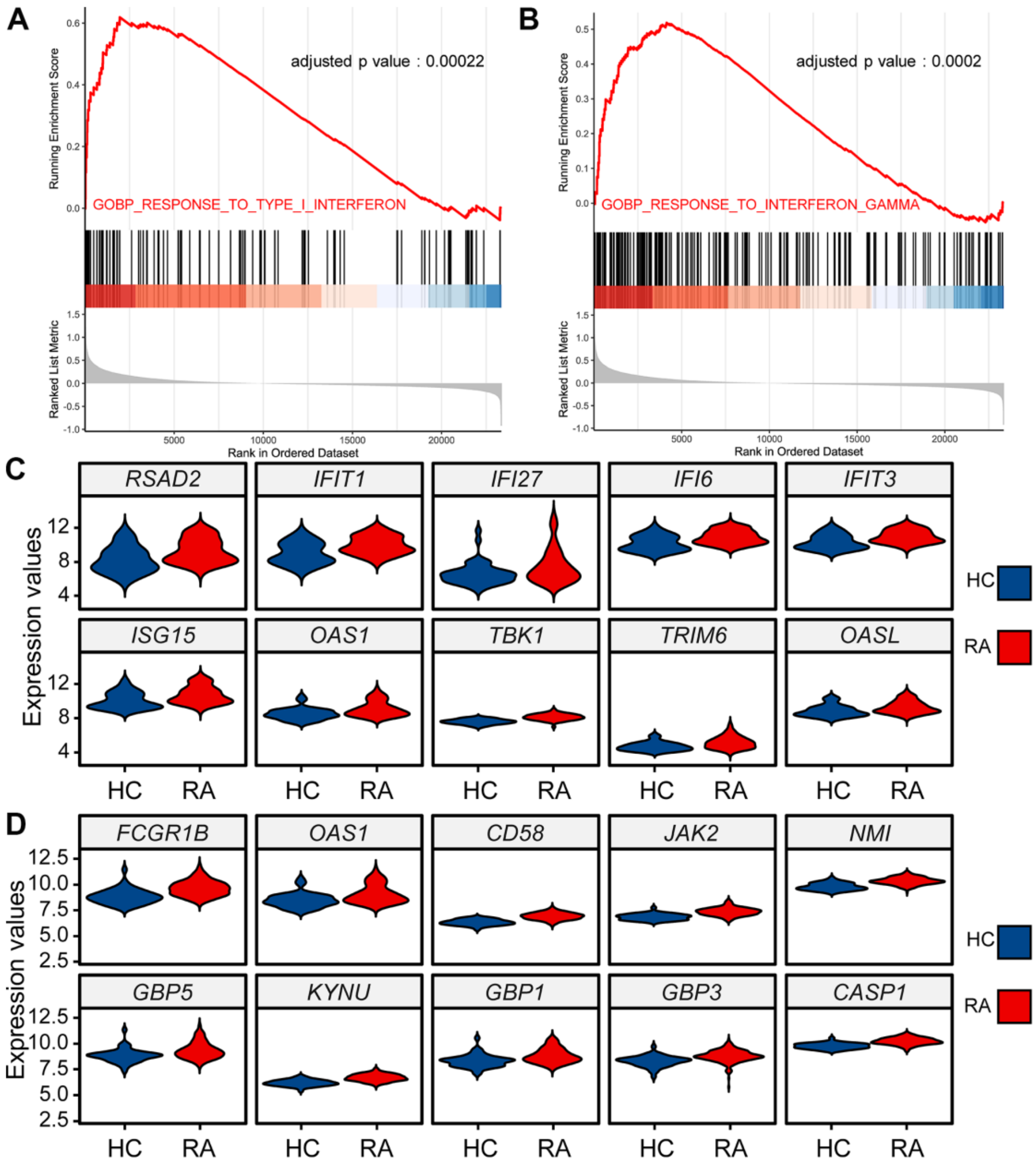

Supplementary Figure 2. Upregulated interferon (IFN)-stimulated signaling pathways and genes in rheumatoid arthritis (RA) peripheral blood. (**A**, **B**) Bar plots of selected results of GSE93272 gene set enrichment analysis (GSEA) indicated upregulated Type I IFN (**A**) and IFN-γ (**B**) stimulated signaling pathways in RA. (**C**) Violin plots of the top 10 upregulated genes in the type I IFN-stimulated signaling pathway. (**D**) Violin plots of the top 10 upregulated genes in the IFN-γ-stimulated signaling pathway. Genes in (**C**, **D**) are ordered according to fold changes, and only genes satisfying adjusted p-value <0.05 are shown.

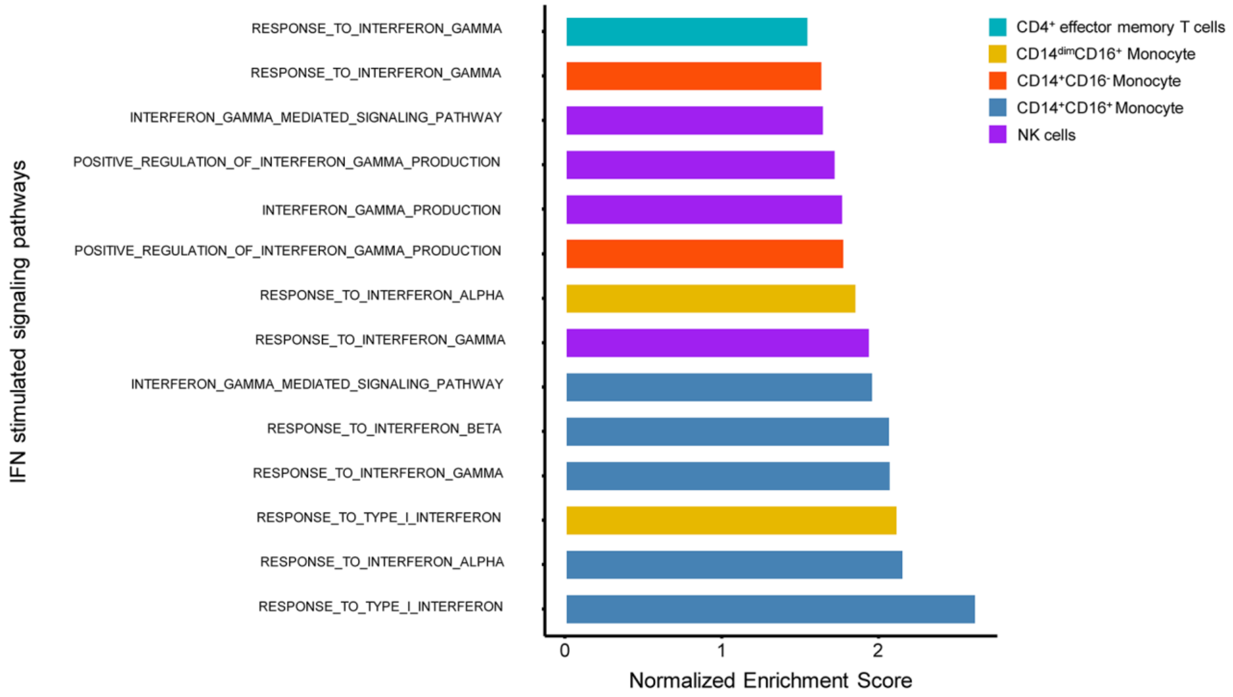

Supplementary Figure 3. Upregulated interferon (IFN)-stimulated signaling pathways in immune cells from rheumatoid arthritis (RA) peripheral blood. Bar plots of selected results of GSE93776 gene set enrichment analysis (GSEA) showing activated IFN-stimulated signaling pathways in RA peripheral blood. The horizontal axis represents the normalized enrichment score. Adjusted p-values of all pathways satisfied were less than 0.05.
